# The RADx Tech Deployment Core: A Model for Academic/Government Based Support of Large-Scale Manufacturing and Implementation of Medical Technologies

**DOI:** 10.1109/OJEMB.2021.3070822

**Published:** 2021-03-26

**Authors:** Brian Walsh, Annette Hosoi, Manuel Kingsley, Susan Moreira, Sreeram Ramakrishnan, Paul Tessier, Nancy Gagliano

**Affiliations:** Innova Group Alpharetta GA 30022 USA; School of EngineeringMassachusetts Institute of Technology 2167 Cambridge MA 02139 USA; Moreira Advisors Northborough MA USA; RxCap Inc. Boston MA USA; Consortia for Improving Medicine with Innovation and TechnologyMassachusetts General Hospital and Harvard Medical School Boston MA 02114 USA; Granite HealthCare Advisors Grantham NH USA

**Keywords:** COVID-19, device testing, NIH, RADx, SARS-CoV-2

## Abstract

This paper explores how the approach, process, and learnings of the RADx^SM^ Tech Deployment Core in its support of manufacturing, deployment, and implementation of medical technologies is creating a replicable model for the future. Initially, the key construct of the RADx Tech Deployment Core was helping companies manufacture, commercialize, and develop a digital infrastructure for the purpose of SARS-CoV-2 testing and reporting. However, the team and RADx Tech leadership soon realized that the larger infrastructure to deploy testing in non-clinical environments was nonexistent and that wrap-around services were required to build the necessary bridge between manufacturing and end users. Furthermore, the unique communities that required testing (e.g., manufacturing plants, transportation hubs, K-12 schools, etc.) had different infrastructure requirements and outsized needs for education and support around testing plan implementation. The Deployment Core, therefore, quickly scaled a team to help to complete the picture and provide guidance to end users and ultimately help shape public policy around a useful data model.

## I. Overview

The RADx^SM^ Tech innovation funnel represents a staged funding approach to efficiently develop, validate, scale, and deploy promising SARS-CoV-2 laboratory-based and point-of-care (POC) diagnostic tests to help contain the COVID-19 global pandemic. This innovative funding and support structure has succeeded in accelerating the typical years-long technology development and commercialization timeline to under 12 months. While Work Package 1 (WP1) of the funnel provides grant funding for rigorous de-risking and accelerated development of clinical-grade prototypes, the contracting vehicle of Work Package 2 (WP2) provides expansive commercial contracts to rapidly scale manufacturing capabilities. This unique funding approach supported the speed required to meet the timeline, as well, as strategic performance gates to assure the projects met milestones.

Designed to address the needs of projects as they moved through the funnel, the Deployment Core was built from scratch under the leadership of the senior author. Once RADx Tech was underway and projects were moving well into the funnel, time was of the essence to build the Core infrastructure, including the additional program management resources necessary to provide technical expertise. Persistent communication channels were designed to ensure rapid and iterative feedback and decision-making as well as the seamless path to market that would be critical for rapid success.

**Fig. 1.**
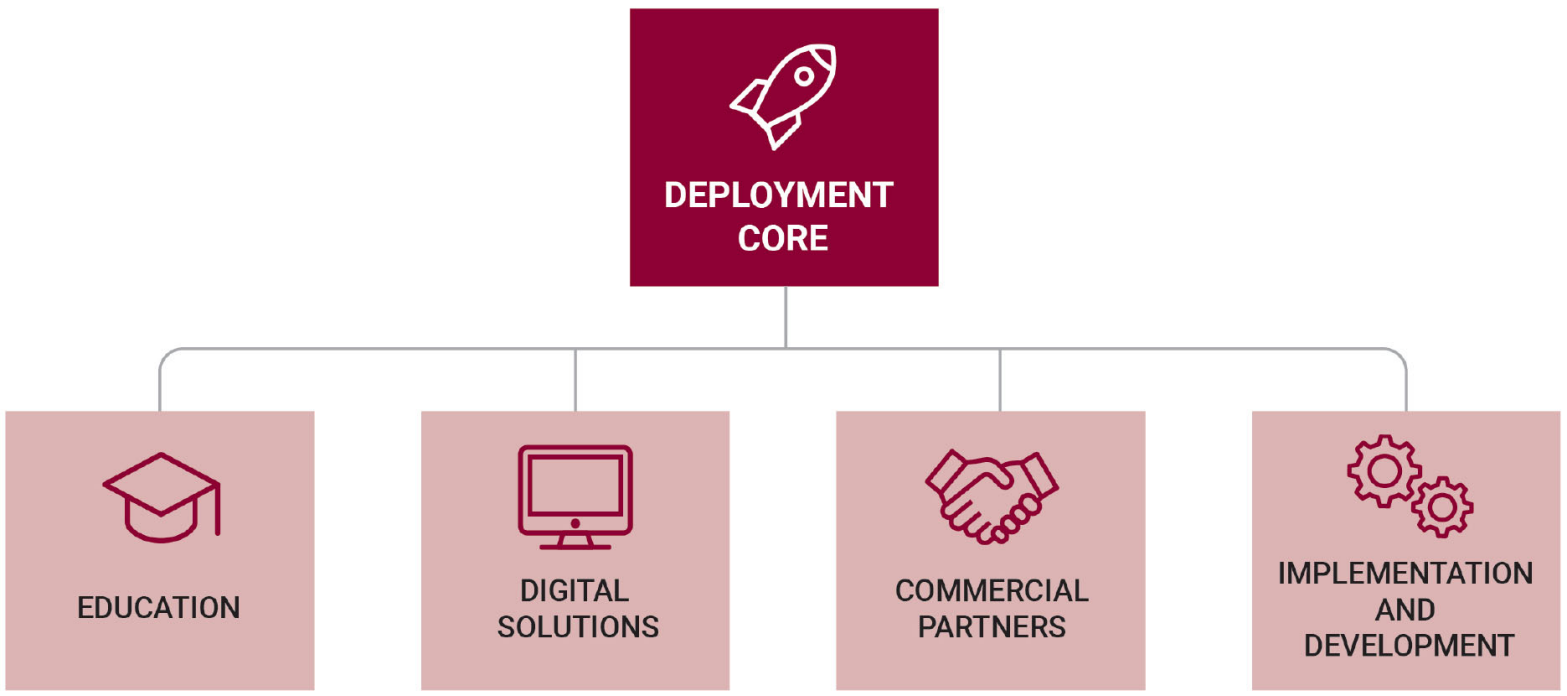
The RADx Tech Deployment Core structure. The Deployment Core was designed to provide technical expertise and coordinated infrastructure to enable market entry of emerging SARS-CoV-2 laboratory-based and point-of-care (POC) diagnostic tests.

At the onset of the initiative, the team held the assumption that the Deployment Core would administer support services for five to ten WP2 companies at or very near the point at which completed products (at the stage of design freeze and verification) were ready for an accelerated scale-up effort. However, many more companies than anticipated were passed through to this phase, and the team soon realized that a majority of these companies lacked the more comprehensive infrastructure required to deploy testing in the non-clinical environments for which their products are intended; wrap-around services were needed to build the necessary bridge between manufacturing and end users. Focused on commercialization, data solutions, implementation, and communications, the Deployment Core is now supporting 60–70 teams for scale-up and manufacturing expansion with budgets totaling more than $480 million, with additional projects in the pipeline.

The Core acted quickly to provide companies with coordinated infrastructure in parallel to enable market entry, including but not limited to procurement and supply chain; manufacturing and development; logistics; distribution; quality management; regulatory; recruiting; reimbursement; and verification/validation.

Furthermore, market research provided numerous insights into the challenges faced by the intended end users of the tests and instruments that RADx Tech seeks to deploy. Non-clinical organizational leaders lacked protocols and guidance around implementing POC testing in their unique environments, where no precedent for diagnostic testing exists. Users have reasonable concerns about testing and screen implementation, cost, feasibility, privacy, and liability. Additionally, test data ultimately need to be collected, stored, and made available to decision makers at local, state, and national levels—without preexisting data policy or infrastructure. The Deployment Core set out to address as best as possible each of these needs and challenges in concert.

## II. Implementation

The Implementation team Within the Deployment Core Acts in a Coordinating Role to Ensure Strong Interaction with RADx Tech regulatory resources, Recruit Outside Supporting Resources Quickly and responsively, and Support design, Clinical testing, manufacturing, Clinical Testing and Other Vital Processes That are Required to Bring an in Vitro Test to market.

### A. Regulatory Processes

Establishing a thoughtful Emergency Use Authorization (EUA) intake process was essential to forming baseline knowledge for the project team. An EUA Testing Intake Form helped the team understand whether the company had any preexisting authorizations, target dates for submission, with what EUA clinical studies the company required external assistance, whether the company had already tested their device in the intended sample type and setting, if a quality management system was in place, and so on. The more known about a project, the more likely a path could be expedited.

The Implementation team also created a formal process for clinical testing that focused on pre-clinical testing, clinical protocols to support the specific tests, and Information for Use (IFU) language and quick reference guides. Clinical testing, at the onset of program, was the responsibility of the CAPCaT Core. This was later modified in the spirit of increasing testing capacity and improving the speed of clinical testing to achieve EUA. The team interviewed and onboarded multiple additional analytical testing labs to perform FDA-required bench testing that would be included in the EUA application. Companies are advised on a proposed path for clinical trials, whether through the Center for Advancing Point of Care Devices in Heart, Lung, Blood, and Sleep Disorders (CAPCaT), the Atlanta Center for Microsystems Engineered Point-of-Care Technologies (ACME), or contract research organizations (CROs).

Of significant impact to commercialization efforts has been the extraordinary relationship between RADx Tech and the FDA. Weekly conversations have been essential to shortening EUA process. RADx Tech regulatory consultants are able to bring high level and project-specific questions directly to the FDA for direct and immediate feedback. This allowed EUA submissions to be accurate and meet FDA expectations right from the beginning. While the FDA did not make any concessions for RADx Tech projects, the relationship clearly expedited the process.

### B. Resourcing and Recruitment

In response to an almost immediate inundation of requests from teams, the Implementation team rapidly enlisted a cadre of expert consultants to spur development on a critical path to commercialization. With a focus on team building, structure, and process, the team rapidly recruited outside supporting resources including commercial team coordinators (CTCs), regulatory consultants, quality management system (QMS) consultants, and project managers, each of whom had significant medical device and manufacturing experience. Additional resources were enlisted as requested by the teams, e.g., development, manufacturing, prototype design improvements, reimbursement, and logistics. Intake and tracking systems were implemented to ensure precise needs were addressed.

However, matching resources to teams came with its own set of challenges. Not all resources were a cultural fit with teams, and re-matches were required. On rare occasions teams were reluctant to accept resources offered, often against their best interest. Those complications were quickly communicated up the ladder, and resources were reallocated. Overall, the vast majority of matches have been successful and critical to the success of the WP2 teams.

### C. Rapid-Response Supply

Procurement was and continues to be an ongoing challenge in light of constrained supply chains. It also quickly became clear that many of the companies have not sufficiently de-risked their supply chain, making it difficult to progress in the timeline in their NIH contract. The Core stood up a rapid-response supply chain team to address shortages of swabs, reagents, robotic chips, automated machinery, and other essential elements. In initial conversations with vendors, it became apparent that more leverage was needed—without a defense priority rating or at least governmental email address it was challenging to elicit adequate response or action from the supply chain. Relationships with multiple government agencies were established, including Dept. of Defense; Assistant Secretary of Preparedness & Response (ASPR); the Joint Acquisition Task Force (JATF-JRAC); the Biomedical Advanced Research and Development Authority (BARDA); and the Federal Emergency Management Agency (FEMA).

Internally, a systematic process was established for the procurement team that included formal intake forms for each company to identify specific needs; tracking forms that were updated daily with status; daily procurement needs review; and standing meetings with government partners to review status and make assignments as well as ad hoc meetings as required.

Resource management and allocation throughout the development pipeline has demonstrated that supporting a seamless path to market is critical for rapid success. A key takeaway from this effort is the importance of recruiting resources in advance of demand and forecasting future need. Leveraging an experienced network of industry professionals reduces the qualification period and increases likelihood of fit in order to onboard at speed.

Another key takeaway for consideration of future initiatives is to make procurement a key pillar of the process, eliminating as much supply chain risk as possible from the onset. Significant effort from the commercialization team was dedicated to establishing a vendor contact list; procuring necessary supplies, in particular for small to mid-size companies, is an ongoing challenge.

## III. Defining and Marketing to User Communities

Essential to the success of deployment is an understanding of the end users to whom these diagnostic tests and tools are being marketed by manufacturers. Working in parallel with other Deployment Core teams, the Marketing team seeks to understand the communities that most stand to benefit from the new technologies. Ultimately these findings help RADx Tech-supported companies think about where and how they are best suited to serve and compete in a marketplace based on the design of their test or instrument.

A challenge was that we were deploying diagnostic technology in settings that have never used diagnostics, such as businesses, K-12 schools, and universities, with the added complication of extreme scarcity. This unprecedented scenario called for in-depth customer discovery to understand how private enterprise views safeguarding their businesses from COVID-19. In particular, small businesses and K-12 schools are most challenged. To this end, the team first reviewed existing surveys that had been made available across multiple sectors. They then conducted in-depth interviews with customers and employers (27); distributors, aggregators, and small suppliers (7); and community organizations/foundations (4). Interviews were conducted as roundtables (utilizing the Zoom platform) with employers (3-6 enterprises represented at a time), who shared their struggles and learnings with regards to testing programs. The Marketing team followed these roundtables with in-depth one-on-one interviews with companies to learn more about their specific disruptions, pain points, and successes. The Marketing team presented companies various models for testing setups and how they might be used, as appropriate.

Findings revealed that dealing with COVID-19 is confusing, costly, complicated, and time consuming for all organizations. Employers have varying protocols and advice to safeguard against COVID-19 and are currently leaning on community-based testing resources. Enterprises struggle with the feasibility and need for testing, questioning what is to be gained from implementing a testing plan (“What will we know that we don't already know”) and how to manage testing in addition to the myriad other protocols currently enforced at state levels. Employers are also deeply concerned with liability and compliance (“Testing in house leads to a lot of privacy issues”).

Among the insights resulting from these survey findings, the lack of clear guidelines, regulation, and financial incentive are impeding testing adoption. Small- to medium-size businesses need solutions that are simple, economical, flexible, and relevant to local circumstances (such as the preponderance of COVID deniers in areas most impacted by misinformation). Company culture also has an outsized impact on how testing is implemented and communicated across the enterprise.

Among its recommendations, the team determined there is a critical need to connect small- to mid-size businesses with COVID test sellers and support, ideally through a platform and/or a centralized clearing house—potentially a public/private partnership—for distributing and administering tests. Organizations also need contact tracing tools and services; liability, reporting, and HIPAA clarity for participants; and government-supported financial safeguards like unemployment and sick leave assistance.

Deployment Core teams are also collaborating with RADx Tech-supported companies to explore how other tests beyond diagnostic tests for the SARS-CoV-2 virus can be incorporated on their innovative technology platforms.

## IV. WhenToTest.org

During initial RADx Tech think tank conversations and presentations related to the blended science of transmission and mitigation strategies, there was an overwhelming consensus among participants regarding the need for access to science-based information and modeling for end users. Market research reinforced the fact that leaders of organizations are unsure how recommendations and testing strategies work together to reduce the risk of COVID-19 outbreaks in their unique environments.

The team partnered with the Massachusetts Institute of Technology Institute for Data, Systems, and Society (IDSS) and the Consortium for Improving Medicine with Innovation & Technology (CIMIT) to provide science-based outputs to justify specific mitigation and testing strategies and demonstrate how COVID-19 prevention and containment efforts can most effectively be combined with the latest testing strategies to minimize the spread of the virus in a specific environment. With a mathematical model in hand — the “When To Test COVID-19 Testing Impact Calculator”—the Deployment Core launched www.WhenToTest.org in early December 2020.

The site and Calculator provide a comprehensive approach to reduce the spread of COVID-19 by comparing common FDA-approved testing and screening options, illustrating cost reduction strategies by implementing best practices, and providing guidance to leaders on how often to test people in their organizations. Users answer five basic questions about mask usage, contact tracing, the degree to which the organization limits unmasked group activities, such as dining, and the percentage of the organization's population that has received a COVID-19 vaccine. Users can also choose to enter cost considerations relating to the time employees are away from their work due to testing and costs associated with completing testing on-site (if applicable).

Calculator outputs reveal how these assumptions impact the recommended testing protocol for the organization, including the estimated testing cost per week and the total number of people to be tested each day. The Calculator assumes that 100% of the population is tested prior to the implementation of an asymptomatic screening protocol. While the baseline goal is to prevent an outbreak, users can modify the default assumptions to meet more assertive goals.

Results are based on five different prototypical testing methods: antigen testing at point-of-care using lateral flow testing (no device); antigen testing at point-of-care (with device); point-of-care molecular (PCR) testing; off-site (central lab) molecular testing; and pooled testing (with immediate off-site PCR follow up). Detailed results are provided for both typical and hotspot conditions, and for additional pooled testing strategies.

K-12 schools have utilized the Calculator to plan their weekly testing cadence and to better understand how to utilize the resources available. Small businesses, nursing homes, prisons, and manufacturing companies have utilized the calculator to help them make decisions around testing types and cadence. The Calculator also helps users see the reduction in testing costs that results from increasing mask wearing, for example, or eliminating communal dining.

Most recently, the team collaborated with the Global Health Crisis Coordination Center (GHC3) and CORE, a Los Angeles-based NGO focused on community-based disaster relief to vulnerable populations, to develop the *When To Test K-12 Playbook*, an educational guide to assist school administrators who are currently conducting or considering conducting in-person classes for their students. This Playbook is a compendium to the WhenToTest.org site and Calculator and provides education leaders with science-based outputs to justify why specific choices and testing strategies will have the greatest return on the school's investment. Prior to release, the Playbook received input from schools, superintendents, nurses, and other stakeholders involved in K-12, protocols, mitigation strategies, and policy.

In addition, the team is currently developing a robust Implementation Guide to help non-clinical organizations implement testing.

## V. Digital Solutions

Access to reliable data will help public health officials and the general public understand where COVID-19 is most prevalent, how far it is spreading, and how quickly it is spreading in each community. Importantly, these data will inform appropriate resource allocation strategies. Moreover, the ability to orchestrate effective contact tracing programs and provide patients with appropriate clinical care is also dependent on the availability of complete, reliable data. A robust data infrastructure will need to have diagnostic results capturing mechanisms, data collection and curation infrastructures, data management and access controls, security and privacy management services, and analytics to enable decision makers at various operational, tactical and strategic decisions. This pandemic is a first of its kind in that we not only must grapple with our understanding of the clinical issues, but also the testing technologies, deployment, scalability, and data logistics in parallel. The complexity of these data challenges are particularly acute for Point Of Care (POC) diagnostic tests which often do not have data capture and reporting integrated within the device.

From purely a technology standpoint, immediate questions include what data to collect (e.g., race and ethnicity), on whose behalf it is being collected, and what is to be done with that data. If a local government will have visibility into that data, will it impact their decisions on whether to open or close schools? Are the data going to be used for contact tracing? Will data inform economic decisions? Defining these gating functions are a priority. The infrastructure we seek to build should provide a framework to, first and foremost, answer these questions as well as the volume and sensitivity of testing data required for informed decision making.

The second essential component of building out this framework is around the policy of who actually owns the data. Are we creating a system where these data are going to be persistent forever? Where will the data be stored? Is it up to the test manufacturers to own, maintain, and report the data, or are test results the patients’ data to share? If the policy concludes that the full reporting of testing data is mandatory, who then decides how to anonymize the data? When we consider a data collection management and storage system for the future, we must start the thread at the point of test manufacturers and clearly define the data that are required of them and the format in which the data should be plugged into, which ideally be a standardized data collection mechanism.

Of concern to companies surveyed in our research is who is responsible, and ultimately liable, for storing the data that is collected by way of POC testing. Another concern is how does knowing an employee's test status potentially violate privacy standards. Once a policy has defined what we will ask stakeholders to do with the access to the data they have, policies for auditing and tracing whether or not they are using the data only for the intended purpose must also be considered. Similar tactical and strategic questions apply to contact tracing. How does one decide which data set is going to be passed on as an artifact to another system dedicated exclusively to contact tracing? While businesses and schools can be encouraged to actively test their populations on site, we must understand this is fraught with challenges and concerns.

During a pandemic, questions around data collection and use lead naturally to analytical questions on how best to use these data to predict who is more likely to contract the virus, i.e., which populations are most susceptible. It is important to understand how quickly predictive and propensity models can be created to better understand where to focus interventions as well as how to refine those interventions for different mechanisms of the spread. In this way we could quickly create some associations or causal relations between existing policies, behaviors, patterns of societies, economic status, and access to healthcare, which could be particularly important in assessing the relative threat posed by different SARS-CoV-2 variants.

In summary, recommendations for the future should be made relative to three decision-making processes: 1) Immediacy: how quickly can we collect patient data and alert contact tracing programs and healthcare systems?; 2) Visibility into how test results are trending in certain locations in order to make decisions around schools, policies, access, transportation, etc.; and 3) Analytics: can we define this spread using defined predictive models? How quickly will we be able to interpret the data so that we can understand where to focus and refine interventions?

Currently this data ecosystem is a moving target; we cannot force an ecosystem to emerge by itself. The Digital Solutions team is working with government agencies to help understand their line of sight and aspirations for defining those data standards and to help them consider all these interfaces together in an effort to shape this ecosystem and ensure that the data of the future is collected, shared, and responded to with equity and efficacy.

## VI. Conclusion

As a model, the Deployment Core efforts reveal how nimble and comprehensive programs and networks are effective in broadening the base of innovations supported by NIH, especially among startup companies and small businesses. Resource management and allocation throughout the development pipeline is critical, as new technologies typically spend years waiting to secure public or private funding for each stage. While NIH has not traditionally provided resources for late-stage development activities such as scale up and manufacturing, RADx Tech has shown that supporting a seamless path to market through public-private partnership and by leveraging industry-wide knowledge can be successful and is critical for rapid success. NIH looks forward to implementing the best practices learned from the RADx Tech experience to accelerate the development and availability of new technologies for additional areas of biomedical research.

## VII Disclaimer

 The views expressed in this manuscript are those of the authors and do not necessarily represent the views of the National Institute of Biomedical Imaging and Bioengineering; the National Heart, Lung, and Blood Institute; the National Institutes of Health, or the U.S. Department of Health and Human Services.

